# The manifestation of anxiety in patients undergoing elective coronary angiography

**DOI:** 10.1192/j.eurpsy.2023.233

**Published:** 2023-07-19

**Authors:** A. Kokalj Palandacic, S. Ucman, B. Novak Sarotar, M. Lainscak

**Affiliations:** 1 University psychiatric clinic Ljubljana; 2 Faculty of Medicine, University of Ljubljana, Ljubljana; 3 General Hospital Murska Sobota, Murska Sobota, Slovenia

## Abstract

**Introduction:**

Prevalence of anxiety disorders in coronary artery disease reaches up to 15% and about half of patients with coronary artery disease have anxiety or depression comorbidity. Prevalence of anxiety in patients undergoing percutaneous coronary intervention ranges from 24% to 72%. Anxiety can be the source of distress and is associated with poor prognosis, impaired health-related quality of life and can cause cardiac dysfunction.

**Objectives:**

We aimed to determine the prevalence of anxiety and its association to coping strategies and personality traits in non-depressed patients undergoing elective coronary angiography, a diagnostic procedure for coronary artery disease. We also aimed to determine the correlation of state anxiety to elective coronary angiography finding and its expression over time.

**Methods:**

This was a single-center, cross-sectional, prospective study. Anxiety was evaluated at four-time points using self-rating questionnaires: 14 days prior to and 2–4h before procedure; 24h after procedure and 6 weeks after discharge. The association between anxiety and psychological variables was assessed by multiple linear regression and by linear mixed effect model.

**Results:**

A total of 259 non-depressed patients were included in the final analysis (median age 65, 32% were female). Prevalence of anxiety was 35% and was higher in patients with avoidance-oriented coping style (*p*<0.001), meanwhile low neuroticism (*p*<0.001) and extrovertive personality trait (p=0.032) were protective factors. Patients that had no intervention (*p*=0.022) or had percutaneous coronary intervention (*p*=0.010) during elective coronary angiography, had lower anxiety than patients in need for coronary artery bypass graft surgery.

**Conclusions:**

Personality traits emotional stability and extroversion are protective factors against anxiety. More than one third of patients experienced clinically significant anxiety before procedure. Our results suggest that recognising anxiety in patients undergoing elective coronary angiography is important. Further on, application of effective interventions for reducing/treating anxiety before or after procedure is needed.

**Disclosure of Interest:**

None Declared

